# *Helicobacter pylori* Activates HMGB1 Expression and Recruits RAGE into Lipid Rafts to Promote Inflammation in Gastric Epithelial Cells

**DOI:** 10.3389/fimmu.2016.00341

**Published:** 2016-09-09

**Authors:** Hwai-Jeng Lin, Fang-Yu Hsu, Wei-Wei Chen, Che-Hsin Lee, Ying-Ju Lin, Yi-Ywan M. Chen, Chih-Jung Chen, Mei-Zi Huang, Min-Chuan Kao, Yu-An Chen, Hsin-Chih Lai, Chih-Ho Lai

**Affiliations:** ^1^Department of Internal Medicine, Division of Gastroenterology and Hepatology, College of Medicine, School of Medicine, Taipei Medical University, Taipei, Taiwan; ^2^Department of Internal Medicine, Division of Gastroenterology and Hepatology, Shuang-Ho Hospital, New Taipei, Taiwan; ^3^Graduate Institute of Basic Medical Science, School of Medicine, China Medical University, Taichung, Taiwan; ^4^Department of Biological Sciences, National Sun Yet-sen University, Kaohsiung, Taiwan; ^5^Department of Medical Research, Genetic Center, School of Chinese Medicine, China Medical University and Hospital, Taichung, Taiwan; ^6^Department of Microbiology and Immunology, Graduate Institute of Biomedical Sciences, Chang Gung University, Taoyuan, Taiwan; ^7^Department of Pediatrics, Molecular Infectious Disease Research Center, Chang Gung Children’s Hospital and Chang Gung Memorial Hospital, Taoyuan, Taiwan; ^8^Department of Medical Biotechnology and Laboratory Science, Chang Gung University, Taoyuan, Taiwan; ^9^Department of Laboratory Medicine, Chang Gung Memorial Hospital, Taoyuan, Taiwan; ^10^Department of Nursing, Asia University, Taichung, Taiwan

**Keywords:** *Helicobacter pylori*, HMGB1, RAGE, cholesterol, interleukin-8

## Abstract

*Helicobacter pylori* infection is associated with several gastrointestinal disorders in the human population worldwide. High-mobility group box 1 (HMGB1), a ubiquitous nuclear protein, mediates various inflammation functions. The interaction between HMGB1 and receptor for advanced glycation end-products (RAGE) triggers nuclear factor (NF)-κB expression, which in turn stimulates the release of proinflammatory cytokines, such as interleukin (IL)-8, and enhances the inflammatory response. However, how *H. pylori* activates HMGB1 expression and mobilizes RAGE into cholesterol-rich microdomains in gastric epithelial cells to promote inflammation has not been explored. In this study, we found that HMGB1 and RAGE expression increased significantly in *H. pylori*-infected cells compared with -uninfected cells. Blocking HMGB1 by neutralizing antibody abrogated *H. pylori*-elicited RAGE, suggesting that RAGE expression follows HMGB1 production, and silenced RAGE-attenuated *H. pylori*-mediated NF-κB activation and IL-8 production. Furthermore, significantly more RAGE was present in detergent-resistant membranes extracted from *H. pylori*-infected cells than in those from -uninfected cells, indicating that *H. pylori* exploited cholesterol to induce the HMGB1 signaling pathway. These results indicate that HMGB1 plays a crucial role in *H. pylori*-induced inflammation in gastric epithelial cells, which may be valuable in developing treatments for *H. pylori*-associated diseases.

## Introduction

*Helicobacter pylori*, a Gram-negative bacterium, colonizes the human stomach and infects more than half of the human population worldwide (1, 2). Persistent infection by *H. pylori* in the stomach induces the production of proinflammatory cytokines, such as interleukin (IL)-1β, IL-6, IL-8, and tumor necrosis factor (TNF)-α (3), which are closely associated with several gastroenterological diseases, including gastritis, peptic ulcer, and gastric adenocarcinoma (4, 5). Moreover, *H. pylori* possesses a set of virulence factors that allow the bacterium to persistently colonize the hostile environment of gastric mucus. These factors include urease, flagella, adhesins, and two major virulence factors, vacuolating cytotoxin A (VacA) and cytotoxin-associated gene A (CagA) (6).

The major components of lipid rafts (also called cholesterol-rich microdomains) are phospholipids, sphingolipids, and cholesterol, which together form tight interactions and create rigid microdomains in the cytoplasm membrane (7). VacA was the first *H. pylori* toxin shown to hijack membrane cholesterol for its own oligomerization and delivery into target cells (8). Translocation, as well as phosphorylation, of CagA into gastric epithelial cells was previously shown to be cholesterol dependent (9). Accordingly, disruption of cholesterol-rich microdomains abolishes the actions of VacA and CagA, mitigating *H. pylori*-associated pathogenesis (9–11). These findings indicate that *H. pylori* orchestrates the exploitation of cholesterol for its intricate infection strategy.

High-mobility group box 1 (HMGB1) is a ubiquitous nuclear protein that stabilizes nucleosomes, enables nicking of DNA, and facilitates transcription (12). HMGB1 has been shown to function as a proinflammatory protein that mediates endotoxin-induced lethality, tissue damage, and systemic inflammation (13, 14). Receptor for advanced glycation end-products (RAGE), a single transmembrane-spanning domain belonging to the immunoglobulin superfamily, serves as a receptor for HMGB1 in the amplification of proinflammatory signaling (15). Interaction of RAGE with HMGB1 triggers mitogen-activated protein kinases (MAPKs) and subsequently activates nuclear factor (NF)-κB (16, 17), thereby stimulating the release of multiple proinflammatory cytokines (18). Moreover, HMGB1 has been implicated in several bacterial diseases that are mediated by inflammatory responses (19–21).

Recently, a study of *H. pylori* revealed that VacA induces programed necrosis of cells, releasing HMGB1, and resulting in a proinflammatory response (22). However, the mechanisms by which *H. pylori* activates HMGB1 expression and mobilizes RAGE into cholesterol-rich microdomains to promote inflammation in gastric epithelial cells have yet to be studied. Therefore, we explored the role of HMGB1 during *H. pylori* infection of gastric epithelial cells. In addition, we investigated whether cholesterol-rich microdomains are involved in the induction of HMGB1 and RAGE expression and the subsequent inflammatory response.

## Materials and Methods

### Reagents and Antibodies

Alexa Fluor 647-conjugated cholera toxin subunit B (CTX-B), Alexa Fluor 488-conjugated goat anti-rabbit IgG, 4′,6-diamidino-2-phenylindole (DAPI), and Lipofectamine 2000 were purchased from Invitrogen (Carlsbad, CA, USA). Anti-HMGB1 (ab18256), anti-RAGE (ab37647), and anti-actin antibodies were purchased from Abcam (Cambridge, MA, USA). Methyl-β-cyclodextrin (MβCD) was purchased from Sigma-Aldrich (St. Louis, MO, USA). Luciferase substrate and β-galactosidase expression vector were purchased from Promega (Madison, WI, USA).

### Bacterial Culture

*Helicobacter pylori* 26695 (ATCC 700392) was recovered from frozen stocks on *Brucella* agar plates (Becton Dickinson, Franklin Lakes, NJ, USA), containing 10% sheep blood (23). Boiled *H. pylori* and bacterial lysates were prepared, as described previously (24).

### Cell Culture

Human AGS cells (ATCC CRL 1739) were cultured in F12 medium (Invitrogen). SCM-1 and TSGH9201 cells were cultured in RPMI 1640 medium (Invitrogen) (24). All culture media were supplemented with 10% fetal bovine serum (HyClone, Logan, UT, USA). For transient transfection, AGS cells were incubated in OPTI-MEM (Invitrogen), 1 μg NF-κB reporter genes, and 1 μl Lipofectamine 2000 for 6 h at 37°C. Transfected cells were then cultured in complete medium for 24 h before further analysis.

### Western Blot Analysis

*Helicobacter pylori*-infected AGS cells were harvested and then boiled in SDS-PAGE sample buffer for 10 min. The protein lysate was then resolved by 10% SDS-PAGE and transferred onto polyvinylidene difluoride membranes (Millipore, Billerica, MA, USA). The membranes were incubated with antibodies against HMGB1 or RAGE at room temperature for 1 h. The blots were washed and then incubated with horseradish peroxidase-conjugated secondary antibody (Millipore). The proteins of interests were detected using the ECL Western Blotting Detection kit (GE Healthcare, Piscataway, NJ, USA).

### Transfection of Small Interfering RNAs

Small interfering RNAs (siRNAs) for RAGE [On-Target*plus* Human AGER (177) siRNA] and scrambled control (sc-37007) were purchased from Thermo Fisher Scientific (Lafayette, CO, USA) and Santa Cruz Biotechnology (Santa Cruz, CA, USA), respectively. AGS cells were transfected with siRNAs (50 nM) by use of Lipofectamine 2000 (Invitrogen) according to the manufacturer’s instructions.

### Quantitative Real-time Reverse Transcription-PCR

Receptor for advanced glycation end-products mRNA levels were analyzed by quantitative real-time PCR using SYBR Green I Master Mix and a model 7900 Sequence Detector System, as described previously (25). The oligonucleotide primers used were corresponded to human RAGE (forward, 5′-CTACCGAGTCCGTGTCTACCA-3′ and reverse, 5′-CATCCAAGTGCCAGCTAAGAG-3′) and glyceraldehyde-3-phosphate dehydrogenase (GAPDH) (forward, 5′-CCCCCAATGTATCCGTTGTG-3′ and reverse, 5′-TAGCCCAGGATGCCCTTTAGT-3′). The program was pre-incubated at 50°C for 2 min and 95°C for 10 min; PCR was performed with 40 cycles of 95°C for 10 s and 60°C for 1 min.

### Reporter Activity Assay

AGS cells were transfected with the NF-κB reporter constructs by using Lipofectamine 2000 prior to infection with *H. pylori* (MOI = 100) (26). Reporter lysis buffer (Promega) was added to the wells, and cells were scraped from the dishes. Equal volumes of luciferase substrate were added to the samples, and luminescence was detected using a microplate luminometer (Biotek, Winooski, VT, USA). Luciferase activity was normalized to transfection efficiency by determining the β-galactosidase activity generated from a co-transfected β-galactosidase expression vector (Promega) (10).

### Determination of IL-8 Production

The concentration of IL-8 was determined by enzyme-linked immunosorbent assay (ELISA), as described previously (27). Briefly, AGS cells were transfected with RAGE siRNA followed by infection with *H. pylori* (MOI = 100) for 6 h. The IL-8 concentration was determined using a sandwich ELISA kit (R&D Systems).

### Immunofluorescence Labeling

AGS cells (2 × 10^5^) were seeded on coverslips in six-well plates and infected with *H. pylori* at an MOI of 100 for 6 h. The cells were fixed with 3.7% paraformaldehyde at room temperature for 1 h and then permeabilized with 0.1% TritonX-100 for 5 min. To label HMGB1 and RAGE, cells were incubated for 30 min with antibodies against HMGB1 and RAGE, followed by probed with Alexa Fluor 488-conjugated goat anti-rabbit IgG and Alexa Fluor 594-conjugated goat anti-rabbit IgG, respectively. The stained cells were analyzed using confocal microscopy (LSM 780; CarlZeiss, Göttingen, Germany) with a 100× objective (oil immersion; numerical aperture, 1.3).

### Analysis of Proteins in Detergent-Resistant Membrane

To isolate detergent-soluble and -resistant fractions, *H. pylori*-infected AGS cells were lysed with ice-cold TNE buffer (25 mM Tris–HCl, pH 7.5, 150 mM NaCl, and 5 mM EDTA), containing 1% (vol/vol) Triton X-100, as described previously (28). Cell lysates were centrifuged at 18,000 × *g* at 4°C for 30 min to separate detergent-soluble and -resistant fractions, as described previously (27). The proteins of interests in each fraction were assessed by Western blot.

### Statistical Analysis

Experimental results are expressed as means ± SEM. The Student’s *t*-test was used to calculate the statistical significance of differences between two groups. The difference was considered significant when *P* < 0.05. Statistical analyses were carried out using SPSS program (version 11.0, SPSS Inc., Chicago, IL, USA).

## Results

### *H. pylori* Infection Induces HMGB1 and RAGE Expression in Gastric Epithelial Cells

**Figure 1:**
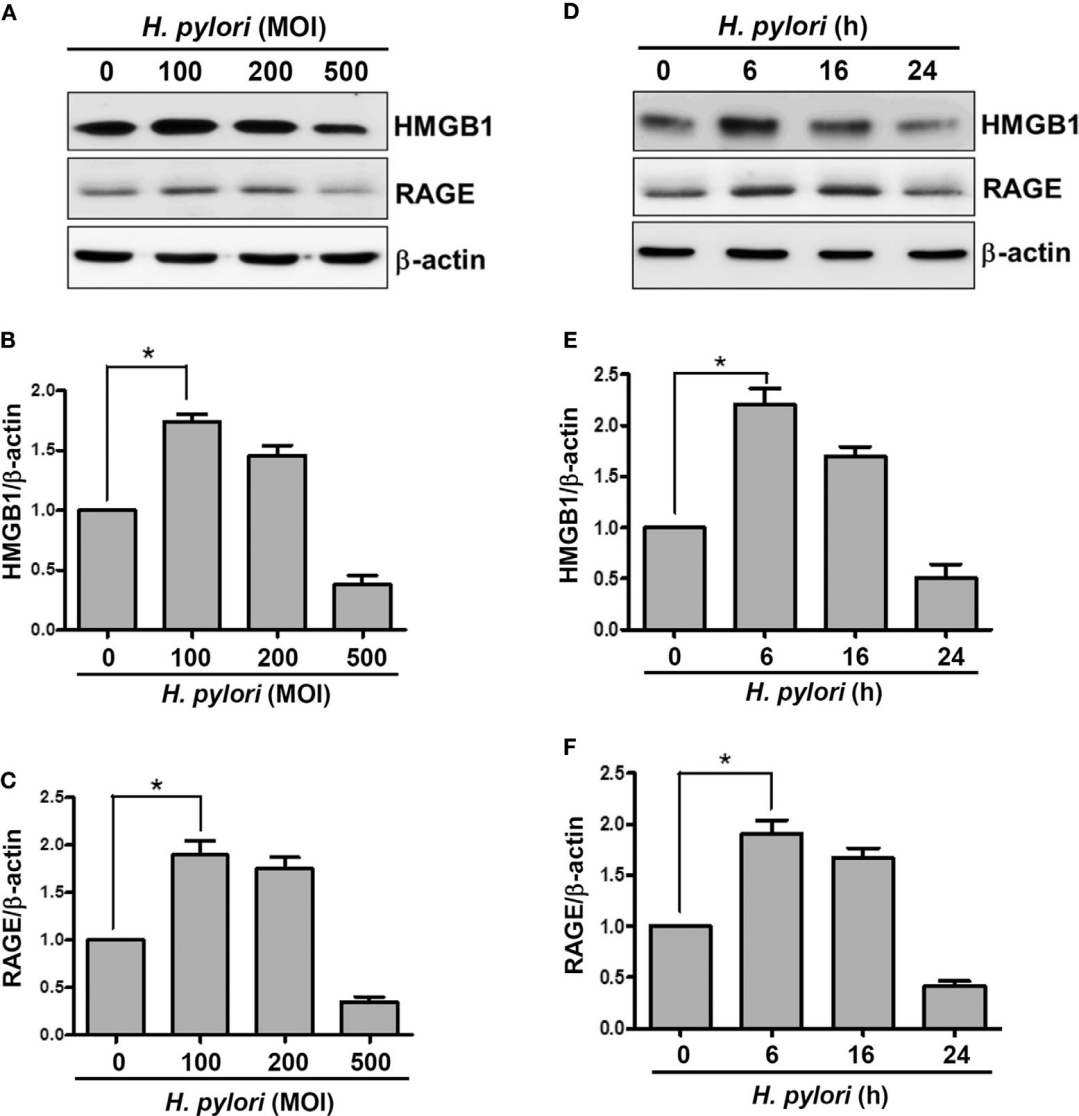
***H. pylori* induces HMGB1 and RAGE expression in gastric epithelial cells**. AGS cells were infected with *H. pylori* for 6 h with various MOIs **(A–C)**, including an MOI of 100 at different time points **(D–F)**. Total cell lysates were prepared to evaluate HMGB1 and RAGE expression by Western blot analysis. Protein expression levels were quantified by densitometric analysis and normalized to β-actin **(B,C,E,F)**. Statistical significance was evaluated by Student’s *t*-test (*P < 0.05).

We first investigated whether *H. pylori* infection induces HMGB1 and RAGE expression in gastric epithelial cells. AGS cells were infected with *H. pylori* at various MOIs (0–500) for 6 h, and the expression levels of HMGB1 and RAGE were determined by Western blot assay. As shown in Figures 1A–C, HMGB1 and RAGE expression levels were markedly increased in cells infected with *H. pylori* at an MOI of 100, whereas they were decreased at higher MOIs of 200 and 500. In addition, AGS cells were infected with *H. pylori* (MOI = 100) for different durations (0–24 h) in parallel. *H. pylori*-induced HMGB1 and RAGE expression peaked with 6 h of infection and decreased after incubation for 16–24 h (Figures 1D–F). These results suggest that *H. pylori* induces HMGB1 and RAGE expression in AGS cells, and that the optimal conditions for infection are an MOI of 100 and incubation for 6 h.

### Live *H. pylori* Is Essential for Enhancing HMGB1 and RAGE Expression in Gastric Epithelial Cells

**Figure 2:**
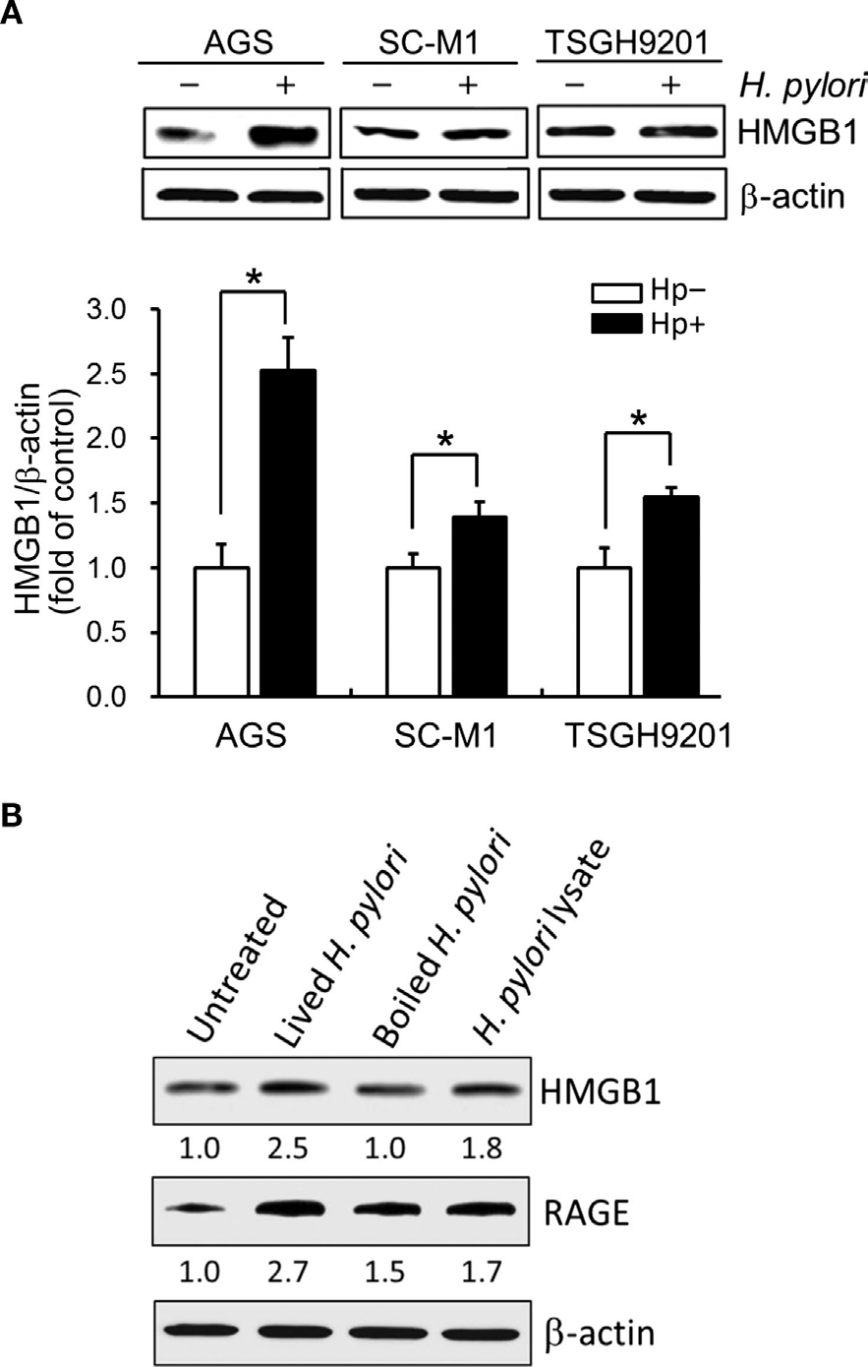
**Live *H. pylori* is essential for enhancing HMGB1 and RAGE expression**. Gastric epithelial cell lines, AGS, SC-M1, and TSGH9201 cells, were infected with *H. pylori* at an MOI of 100 for 6 h. **(A)** Cells from these cells lines were uninfected or infected with *H. pylori* (MOI = 100) for 6 h. Cell lysates were prepared to analyze HMGB1 expression by Western blot. Protein expression levels were quantified by densitometric analysis and normalized to β-actin. Statistical significance was evaluated by Student’s *t*-test (*P < 0.05). **(B)** AGS cells were untreated or treated with live *H. pylori* or heat-killed *H. pylori* (boiled *H. pylori*) at an MOI of 100, or crude extracts prepared from *H. pylori* (*H. pylori* lysate). Cell lysates were prepared to measure HMGB1 and RAGE protein expression by Western blot, with β-actin was used as the protein loading control. The expression level of each protein was quantified by signal intensity, and the respective value is indicated at the bottom of each lane.

We then explored whether increased HMGB1 expression could be seen in AGS and two other gastric epithelial cell lines (SC-M1 and TSGH9201). As shown in Figure 2A, the expression levels of HMGB1 were significantly elevated in the three *H. pylori*-infected gastric epithelium-derived cell lines. AGS cells were found to be the most susceptible; therefore, this line was chosen for the following investigations. We next analyzed the effects of live or killed *H. pylori* with the ability to elicit HMGB1 and RAGE expression in AGS cells. Live bacteria, boiled bacteria (heat-killed), and bacterial lysates (crude extracts) were examined for their capacity to induce HMGB1 and RAGE. As shown in Figure 2B, HMGB1 and RAGE expression in AGS cells in response to live *H. pylori* increased significantly, whereas boiled bacteria and bacterial lysates only slightly increased the expression of HMGB1 and RAGE in these cells. Our data showed that the expression levels of HMGB1 and RAGE were elevated in *H. pylori*-infected AGS cells and that live bacteria were required.

### *H. pylori*-Induced RAGE Expression Is Elicited by HMGB1

**Figure 3:**
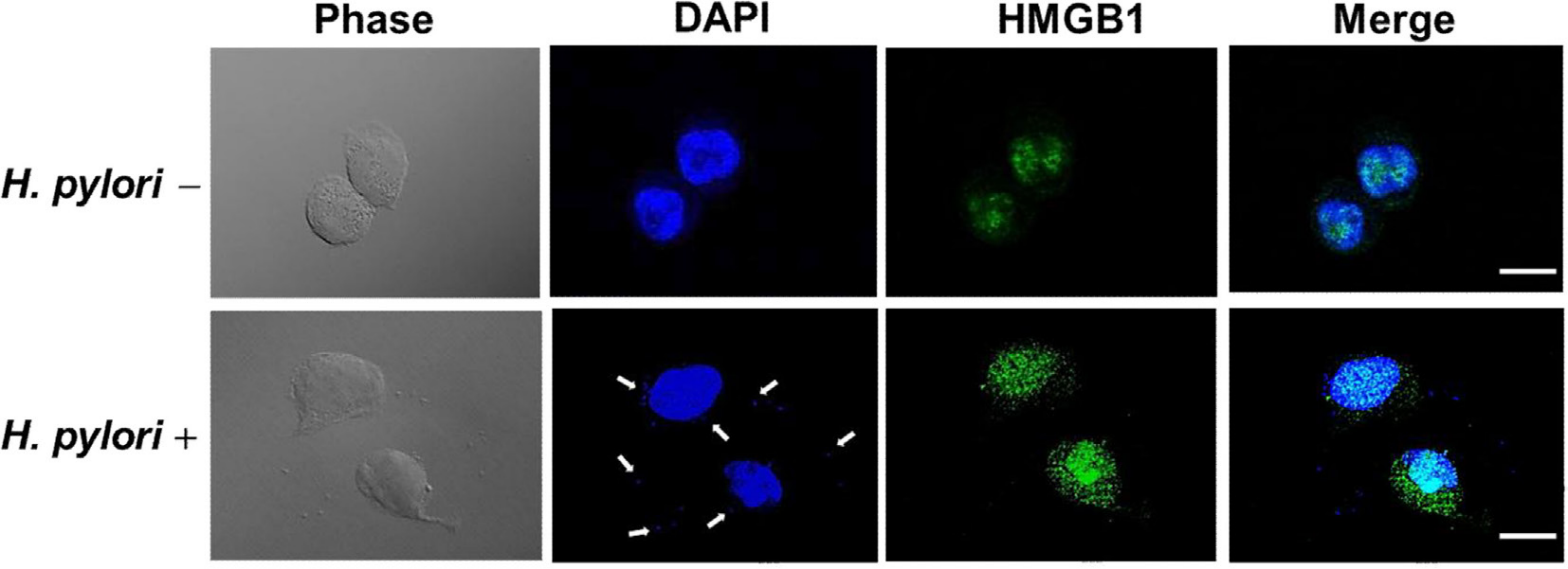
**HMGB1 expression in response to *H. pylori* infection**. AGS cells were uninfected or infected with *H. pylori* (MOI = 100) at 37°C for 6 h. Cells were fixed and probed with antibody against HMGB1 (green) or stained with DAPI (blue) to visualize cell nuclei and *H. pylori* (arrows). The stained samples were analyzed by confocal microscopy. Scale bars, 10 μm.

**Figure 4:**
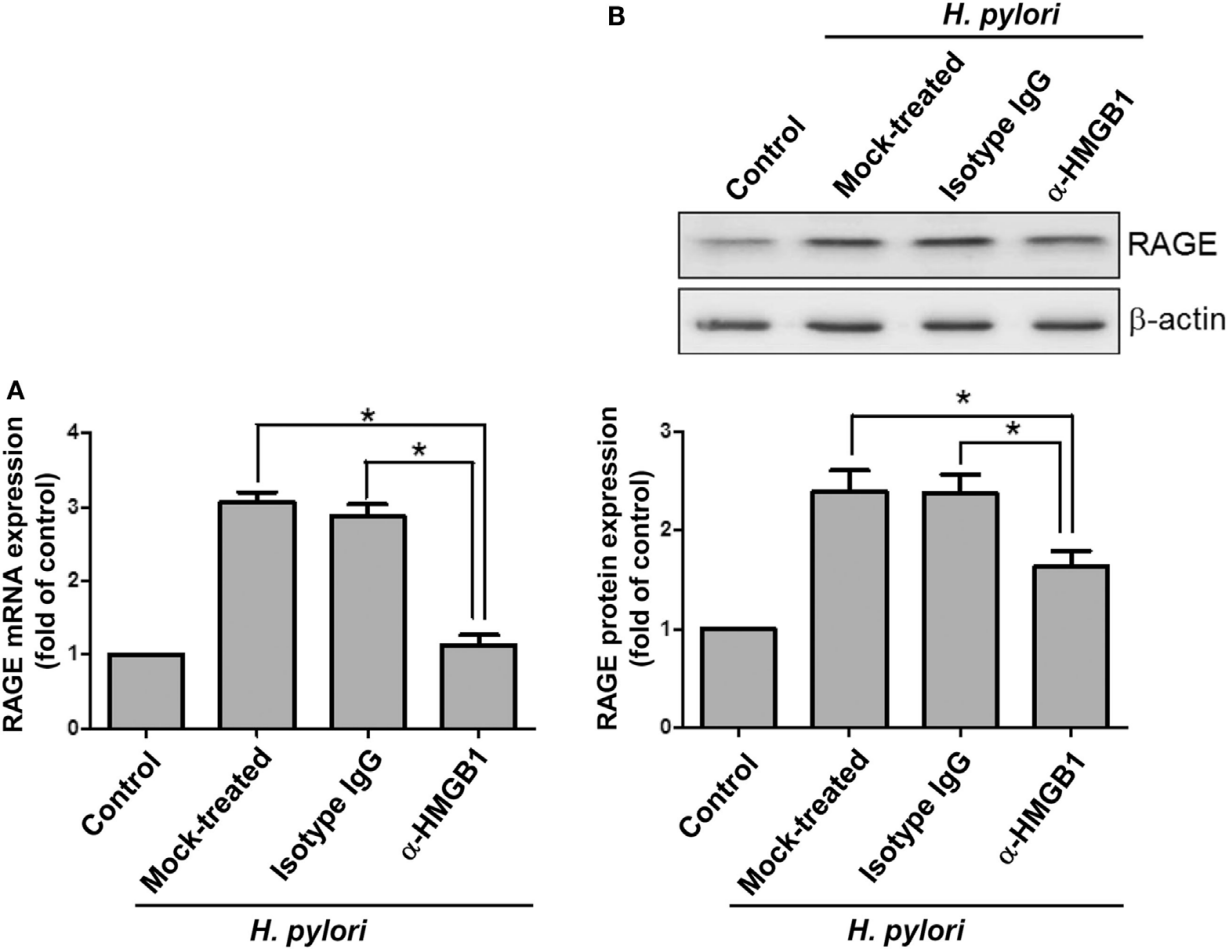
**HMGB1 is crucial for RAGE expression in *H. pylori*-infected cells**. AGS cells were untreated or pretreated with 1 μg/ml of isotype IgG or anti-HMGB1 at 37°C for 30 min and then infected with *H. pylori* at an MOI of 100 for 6 h. RAGE mRNA and protein expression levels were measured by **(A)** quantitative real-time PCR and **(B)** Western blot analysis, respectively. Results are expressed as means ± SDs. *P < 0.05.

Confocal microscopy was used to observe HMGB1 expression in AGS cells. As shown in Figure 3, without *H. pylori*, the image showed faint HMGB1 staining in cell nuclei. In contrast, the distribution of fluorescence clearly showed that HMGB1 localized in both the nucleus and the cytoplasm of cells upon *H. pylori* infection. We then analyzed RAGE expression in response to *H. pylori*-induced HMGB1. AGS cells were mock-treated or -pretreated with isotype IgG or neutralizing antibody against HMGB1 (α-HMGB1) for 30 min and then incubated with *H. pylori* for 6 h. As shown in Figure 4, blocking of HMGB1 by α-HMGB1 significantly reduced *H. pylori*-induced RAGE mRNA and protein levels, whereas this mock-treated cells or cells treated with isotype IgG showed no such effect. These results indicate that *H. pylori* infection induces HMGB1 expression, which in turn elicits the production of RAGE in gastric epithelial cells.

### Silencing RAGE mRNA Ameliorates *H. pylori*-Induced Inflammation

**Figure 5:**
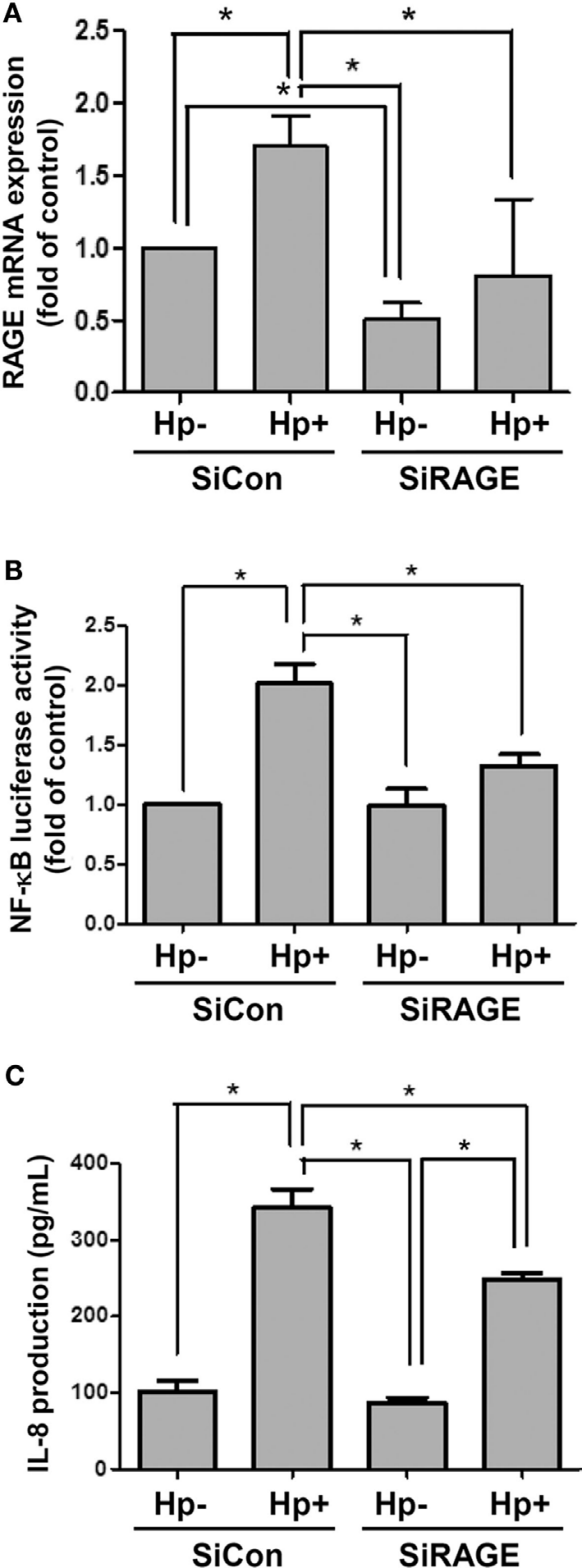
**Knocking down RAGE reduces NF-κB promoter activity and IL-8 production in *H. pylori*-infected AGS cells**. Cells were transfected with control siRNA (SiCon) or RAGE siRNA (SiRAGE) for 24 h prior to infection with *H. pylori* (MOI = 100) for 6 h. **(A)** The RAGE mRNA level was determined by quantitative real-time PCR. **(B)** Cells were co-transfected with SiRAGE and NF-κB/wt luciferase reporter for 24 h and cultured with *H. pylori* (MOI = 100) for an additional 6 h. NF-κB promoter activity was analyzed by luciferase reporter assay. **(C)** The level of IL-8 in the culture supernatant was determined using a standard ELISA. Results were expressed as means ± SDs. *P < 0.05.

AGS cells were then transfected scrambled control siRNA (SiCon) or RAGE siRNA (SiRAGE) for 24 h following incubation with *H. pylori* for 6 h. A quantitative real-time PCR analysis showed that SiRAGE transfection significantly reduced the level of RAGE mRNA when compared to SiCon transfection (Figure 5A). Additionally, *H. pylori*-induced RAGE mRNA expression was markedly suppressed by transfection with siRAGE. We therefore analyzed whether silencing RAGE decreased NF-κB promoter activity and IL-8 production in *H. pylori*-infected cells. Cells were co-transfected with SiRAGE and an NF-κB/wt luciferase reporter prior to incubation with *H. pylori* for 6 h and then subjected to luciferase activity assay. Culture supernatants were harvested to evaluate IL-8 production by ELISA. Our data showed that both NF-κB promoter activity and IL-8 production were significantly reduced by knocking down RAGE in cells infected with *H. pylori* (Figures 5B,C). These results confirm *H. pylori*-induced inflammation in response to reciprocally elicited HMGB1 and RAGE expression.

### Mobilization of RAGE into Cholesterol-Rich Microdomains by *H. pylori* Induces IL-8 Production

**Figure 6:**
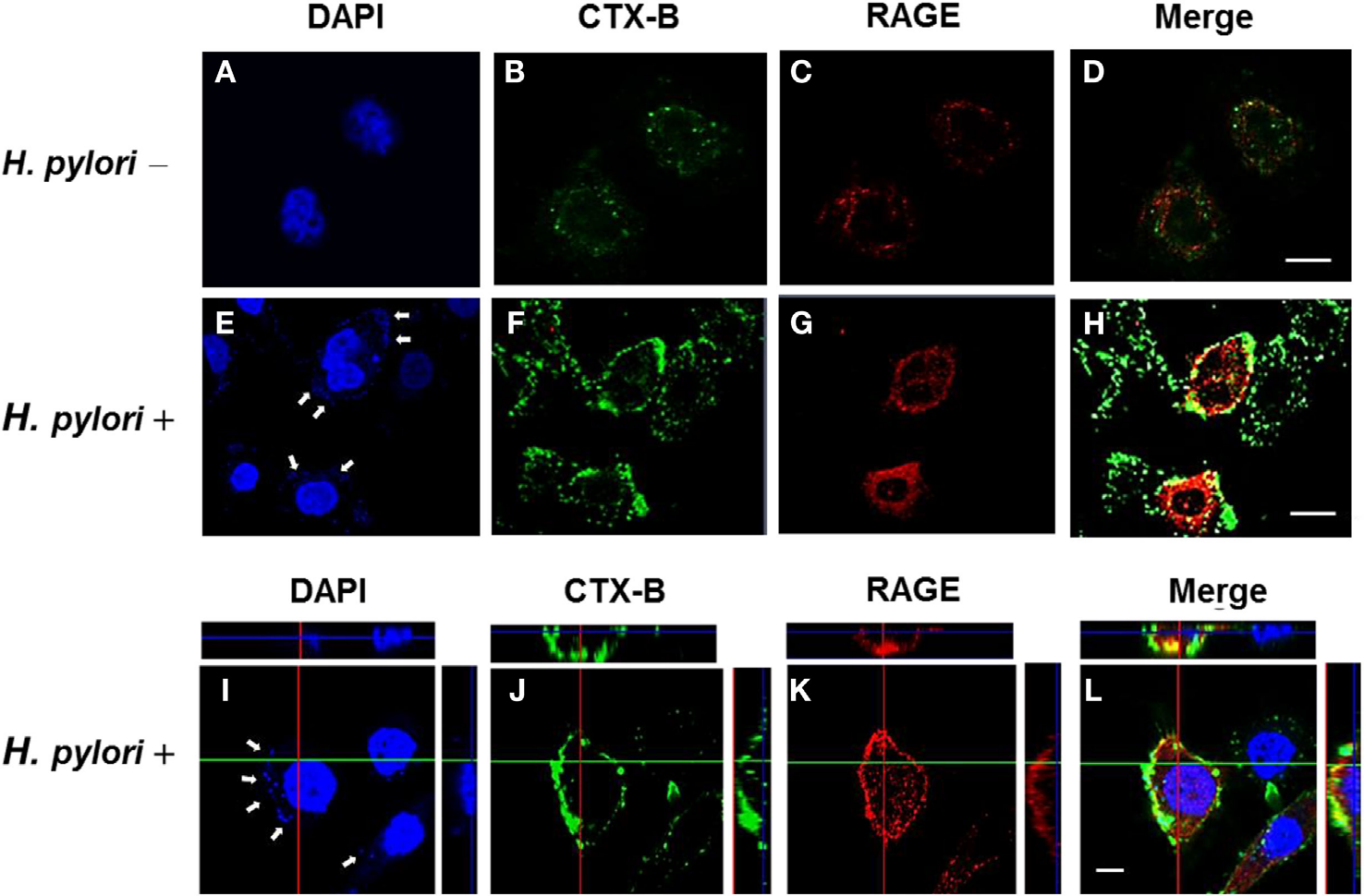
**Mobilization of RAGE into lipid rafts at sites of *H. pylori* infection**. AGS cells were uninfected or infected with *H. pylori* (MOI = 100) for 6 h. Cells were fixed and stained with DAPI (blue) **(A,E,I)** to visualize *H. pylori* (arrows) and cell nuclei, with Alexa Fluor 488-conjugated cholera toxin subunit B (CTX-B) to visualize GM1 (green) **(B,F,J)**, or with antibody against RAGE (red) **(C,G,K)**, and then the merged images were observed by confocal microscopy **(D,H,L)**. Merged confocal *z*-section images **(I–L)** show bacteria colocalized with RAGE and CTX-B (cyan). Bars, 10 μm.

The involvement of cholesterol-rich microdomains in the induction of RAGE by *H. pylori* infection was explored next. The colocalization of RAGE with CTX-B, a raft-associated molecule that binds to the ganglioside GM1, was clearly observed around the cytoplasmic membrane in *H. pylori*-infected cells (Figures 6E–H); this effect was minimal in uninfected cells (Figures 6A–D). The merged images were then analyzed by confocal microscopy *z*-section. As shown in Figures 6I–L, the adhered bacteria (arrows) clearly appeared to colocalize with RAGE and CTX-B in the cytoplasmic membrane. These results indicate that the recruitment of RAGE into membrane rafts occurs in response to *H. pylori* infection.

**Figure 7:**
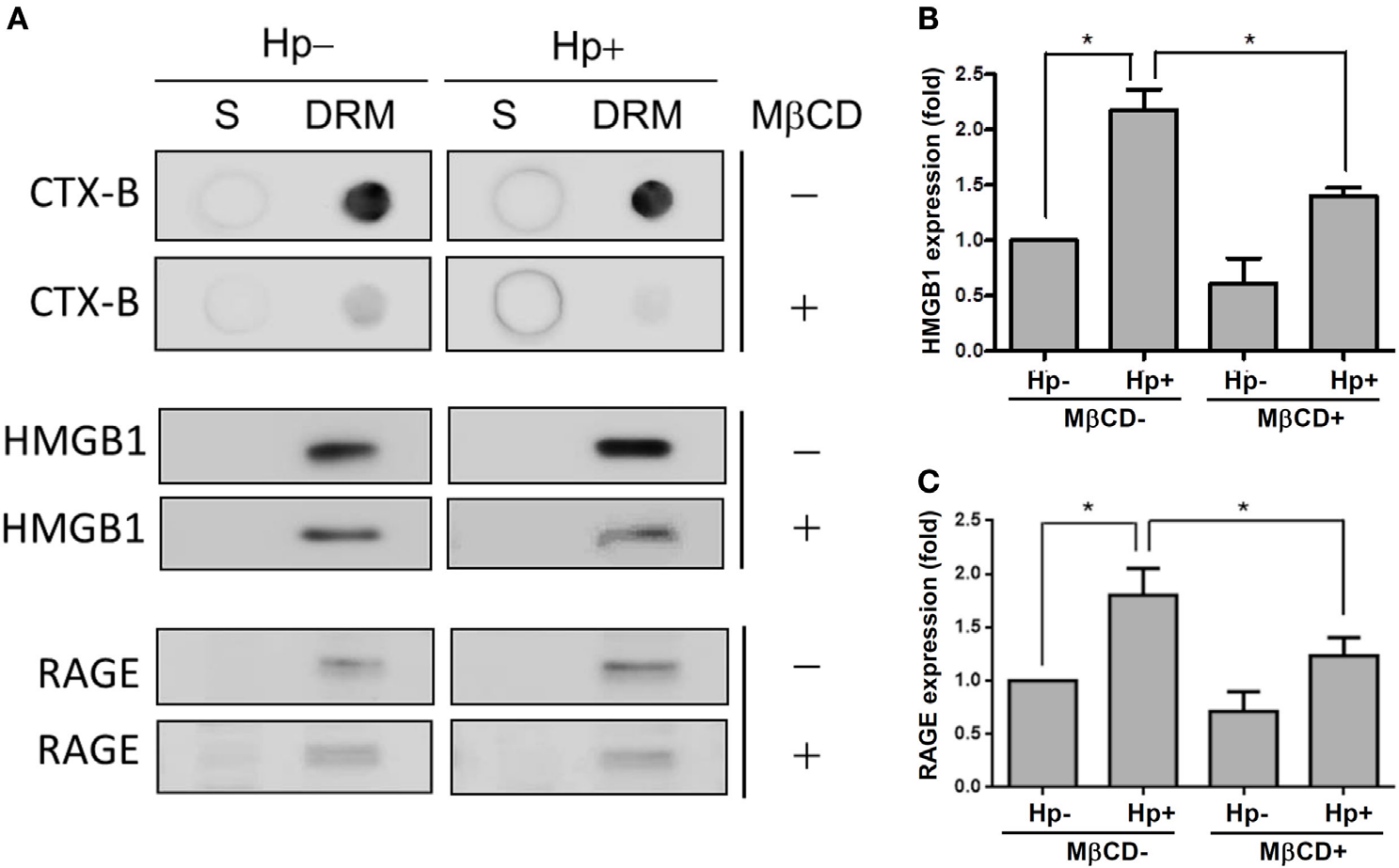
**Role of cholesterol-rich microdomains in *H. pylori*-induced HMGB1 and RAGE expression**. AGS cells were untreated or pretreated with 5 mM MβCD at 37°C for 1 h. Cells were then washed and infected with *H. pylori* at an MOI of 100 for 6 h. **(A)** Detergent-resistant membrane (DRM) and detergent-soluble (S) fractions were prepared and subjected to cold detergent extraction using 1% Triton X-100 at 4°C followed by centrifugation. Each fraction was analyzed by dot blot or Western blot using cholera toxin subunit B (CTX-B) conjugated to horseradish peroxidase or antibodies against HMGB1 and RAGE, respectively. Protein expression levels of **(B)** HMGB1 and **(C)** RAGE were quantified by densitometric analysis (*P < 0.05).

We further investigated whether *H. pylori*-induced HMGB1 and RAGE expression required lipid raft integrity. Western blot analysis showed that CTX-B was enriched in the detergent-resistant membrane (DRM) fraction (Figure 7A), whereas disrupting lipid rafts with MβCD reduced the presence of CTX-B in the DRM. During *H. pylori* infection, HMGB1 and RAGE were abundant in the DRM fraction. Moreover, treatment of cells with MβCD led to a significant reduction in *H. pylori*-induced HMGB1 and RAGE expression in the DRM (Figures 7B,C), suggesting that cholesterol-rich microdomains play an important role in *H. pylori*-triggered HMGB1 and RAGE expression.

**Figure 8:**
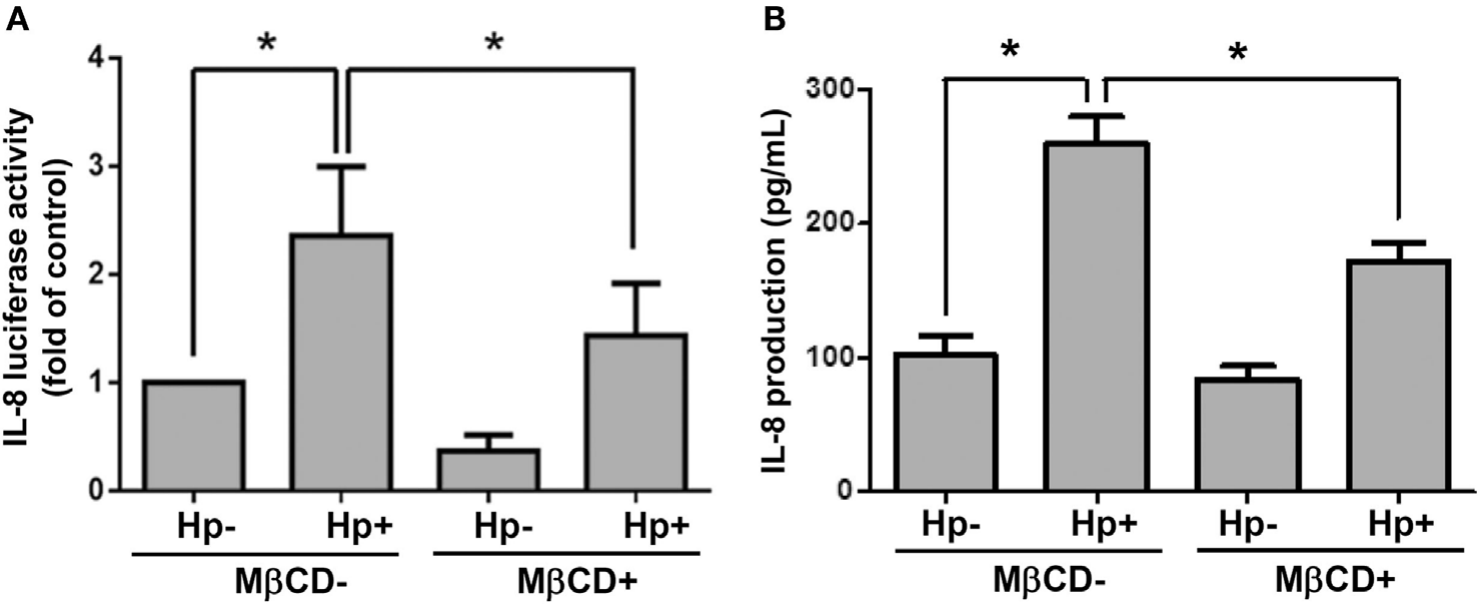
**Disruption of lipid rafts reduces *H. pylori*-induced IL-8 production**. AGS cells were transfected with an *IL-8* luciferase reporter in the absence or presence of 5 mM MβCD prior to infection with *H. pylori* (MOI = 100) for 6 h. **(A)** Cell lysates were subjected to luciferase activity assay to assess *IL-8* promoter activity. **(B)** IL-8 secretions in the cell culture supernatants were assessed by ELISA. Statistical significance was evaluated by Student’s *t*-test (*P < 0.05).

We next examined whether cholesterol-rich microdomains were essential for *H. pylori*-induced IL-8 production. AGS cells were untreated or pretreated with MβCD and then incubated with *H. pylori* for 6 h. Results showed that MβCD treatment significantly suppressed *IL-8* promoter activity in *H. pylori*-infected cells (Figure 8A). Similarly, *H. pylori*-induced IL-8 production in cells was markedly reduced when cholesterol-rich microdomains were disrupted by MβCD (Figure 8B). Taken together, results from this study demonstrate that depletion of cholesterol inhibits the mobilization of RAGE into cholesterol-rich microdomains, thereby mitigating *H. pylori*-induced inflammation.

## Discussion

Infection with *H. pylori* is associated with sustained inflammation, which may lead to severe gastric diseases (5). Previous studies have indicated that HMGB1 can be secreted by *H. pylori* VacA-treated cells, which then underwent necrosis, inducing a proinflammatory response (22). Moreover, *H. pylori* infection increases the expression of RAGE, which subsequently interacts with its ligand HMGB1, and is believed to amplify the inflammation cascade (29). Despite the fact that the interaction of HMGB1 and RAGE can be linked to necrosis and a proinflammatory response in cells (30), the detailed mechanism by which *H. pylori* induces HMGB1 and RAGE expression and triggers IL-8 secretion to promote inflammation of gastric epithelial cells remains unclear. To elucidate the direct mechanical effects of bacterial infection, we employed antibody neutralization of HMGB1 and siRNA for RAGE and demonstrated that *H. pylori*-induced RAGE following the elevation in HMGB1 levels. Furthermore, RAGE was mobilized into lipid rafts, which contributed to the induction of NF-κB activation and IL-8 production during *H. pylori* infection. Notably, depletion of cholesterol diminishes *H. pylori*-induced signaling, confirming the recruitment of RAGE into lipid rafts by *H. pylori* to promote inflammation in gastric epithelial cells.

High-mobility group box 1 has been recognized as a damage-associated molecular pattern (DAMP), and it has been implicated in several bacterial diseases, including inflammatory lung injury (20), pneumonia (19), sepsis (31), and keratitis (32). Accumulating evidence indicates that HMGB1 functions as an alarmin, forming immune stimulatory complexes with chemotactic factors that promote the migration of leukocytes, activation of lymphoid cells, and augment the inflammatory response (30, 33, 34), which correlate with severity of infection (21). RAGE, a ligand for HMGB1, is involved in activating NF-κB and stimulating proinflammatory factors (35). Treatment of mice with neutralizing α-HMGB1 reduced the bacterial burden and ameliorated tissue injury (20, 32). Similarly, blocking HMGB1 reduced *H. pylori*-elicited RAGE expression, resulting in the attenuation of NF-κB activation and thereby mitigating inflammation in gastric epithelial cells. Our findings are in accordance with previous studies with other bacteria, indicating a potential pathogenic role for HMGB1 and RAGE.

In this study, we showed that *H. pylori* infection elicits HMGB1 and RAGE expression, which enhances IL-8 production. In contrast, silencing RAGE appears to reduce *H. pylori*-mediated NF-κB and IL-8 activities. However, NF-κB and IL-8 activities were still greater in siRAGE-transfected cells infected with *H. pylori* than in transfected cells that were uninfected. These results suggest that there are diverse receptors and ligands for HMGB1 and RAGE that interact and contribute to *H. pylori*-induced inflammation. For instance, HMGB1 is able to trigger a proinflammatory response by interacting with either IL-1β, CXCL12 to form immune stimulatory complexes, or several cell surface receptors, including RAGE, toll-like receptor 2 (TLR2), and TLR4 (15, 36). Our recent findings support the explanations that infection of gastric epithelial cells with *H. pylori* induces TLR4/MD-2 expression, which contributes to the inflammatory response (27). On the other hand, RAGE can bind ligands other than HMGB1, including amyloids and members of the S100 protein family (37, 38). Understanding the interactions other than those of HMGB1 and RAGE is required to further investigation the molecular patterns involved in immune sensing following infection with *H. pylori*.

Damage-associated molecular patterns are endogenous danger signals that have been identified, including HMGB1, S100A8/9, IL-1α, and IL-33/ST2 (39–41). Activation of HMGB1 signal is mediated by several pattern-recognition receptors (PRPs), such as RAGE and toll-like receptors (TLRs), that are important for *H. pylori*-induced inflammation has been revealed in our and other studies (24, 29, 42). Similar to HMGB1, IL-1α and IL-33/ST2 also are types of alarmins, which are abundantly expressed in epithelial and endothelial cells (43, 44). Expression of IL-1α and IL-33/ST2 has been reported in several bacterial infectious diseases. For example, IL-1α production was essential for the early recruitment of neutrophils to the lungs infected with *Legionella pneumophila* (45). In patients with *Staphylococcus aureus* infection on the skin, IL-33 is markedly increased as compared to the healthy controls and suggested that IL-33 possesses antimicrobial and wound-healing effects (46). However, limited reports indicated that IL-1α and IL-33/ST2 can be upregulated in cells treated with the virulence factors from *H. pylori* (47, 48), but their role in *H. pylori*-induced pathogenesis is ill defined. Although these DAMPs have been found to be associated with necroptosis, which is an important process for induction of inflammatory diseases (41), the exact role in *H. pylori*-induced inflammation remains to be investigated.

This study presents a model of the early *H. pylori*-induced gastric epithelial cell inflammatory response. The expression of HMGB1 and RAGE was only tended to increase with infections for 6 h. After incubation for a longer time, the expression levels of HMGB1 and RAGE were diminished. This trend can also be seen in infections with *S. aureus* and other Gram-negative bacteria in mouse models (19, 20, 49). One possible explanation for this observation is that cytokine production was substantially reduced at a time point later than 6 h, which may result in a reciprocal reduction in HMGB1 release and amelioration of bacteria-induced pathogenesis.

Although an inflammatory response with the recruitment of leukocytes is crucial for eradicating intracellular pathogens, prolonged activation of neutrophils may result in serious tissue damage in the stomach (50). IL-8 is recognized as one of the most important chemokines that cause neutrophils to infiltrate into sites of bacterial infections (51). Moreover, HMGB1 is reported to be a chemoattractant for neutrophils during inflammation (52). In this study, we showed that *H. pylori* exploits cholesterol to induce inflammation through activation of the HMGB1–RAGE–IL-8 axis. Silencing RAGE significantly attenuated *H. pylori*-induced NF-κB activation and IL-8 production. Our results, combined with the findings of others, indicate that HMGB1 might be a key target for the development of therapeutic agents against *H. pylori*-induced inflammation.

Although our study has demonstrated that *H. pylori* exploits cholesterol to induce inflammation through activation of the HMGB1–RAGE–IL-8 axis, the limitation of this work is that it lacks *in vivo* data. It has been reported that the human serum HMGB1 levels are significantly and sequentially increased during gastric cancer progression (53). Similarly, in a previous study, the HMGB1 expression in gastric cancer tissues was increased as compared to that in non-cancerous tissues (54). Moreover, a markedly higher percentage of RAGE expression was found in *H. pylori*-infected biopsies with dysplasia or *in situ* carcinoma as compared to that in the control groups (55). Most importantly, it has been proven that overexpressed HMGB1 enhances IL-8 secretion in tumor cells and over-secreted IL-8 promotes EMT activation in gastric cancer cells (56). Accordingly, blocking HMGB1 suppresses gastric cancer cell proliferation, whereas inducing IL-8 reverses this anti-tumor effect. These findings demonstrated the role of HMGB1 and RAGE as inducers of inflammation in the context of gastric cancer and suggested that they could be attractive targets for diagnosis and therapy of patients with incipient gastric cancer. Thus, our results supported by evidence in the existing literature, and further revealed that HMGB1–RAGE–IL-8 axis may play an important role in clinical features of *H. pylori*-induced inflammation. Although the present work did not include human studies, we believe that it deserves to be explored *in vivo* and that will definitely fill a gap in the translational research.

Here, we report that *H. pylori*-induced RAGE expression follows HMGB1 production. Our study shows that *H. pylori* infection mobilizes RAGE into cholesterol-rich microdomains, which contributes to NF-κB activation and IL-8 secretion. Furthermore, we elucidate the role for reciprocal, cholesterol-dependent interactions of HMGB1, and RAGE in IL-8 production during the early phase of *H. pylori*-induced inflammation in gastric epithelial cells.

## Author Contributions

Conception or design of this work: H-JL, H-CL, and C-HL. Experimental study: F-YH, W-WC, C-HL, and C-JC. Data analysis and interpretation: Y-JL, Y-YC, M-ZH, M-CK, and Y-AC. Writing the manuscript: H-JL, H-CL, and C-HL. Final approval: all authors.

## Conflict of Interest Statement

The authors declare that the research was conducted in the absence of any commercial or financial relationships that could be construed as a potential conflict of interest.

## References

[1] Parsonnet J. *Helicobacter* *pylori*. Infect Dis Clin North Am (1998) 12:185–97. 10.1016/S0891-5520(05)70417-7 9494838

[2] Marshall B *Helicobacter pylori*: 20 years on. Clin Med (2002) 2:147–52. 10.1007/s102380200021 PMC4952378 11991099

[3] Harris PR Smythies LE Smith PD Dubois A. Inflammatory cytokine mRNA expression during early and persistent *Helicobacter pylori* infection in nonhuman primates. J Infect Dis (2000) 181:783–6. 10.1086/315257 10669377

[4] Cover TL Blaser MJ. *Helicobacter pylori* in health and disease. Gastroenterology (2009) 136:1863–73. 10.1053/j.gastro.2009.01.073 19457415 PMC3644425

[5] Wroblewski LE Peek RM Jr Wilson KT. *Helicobacter pylori* and gastric cancer: factors that modulate disease risk. Clin Microbiol Rev (2010) 23:713–39. 10.1128/CMR.00011-10 20930071 PMC2952980

[6] Amieva MR El-Omar EM. Host-bacterial interactions in *Helicobacter pylori* infection. Gastroenterology (2008) 134:306–23. 10.1053/j.gastro.2007.11.009 18166359

[7] Ikonen E. Roles of lipid rafts in membrane transport. Curr Opin Cell Biol (2001) 13:470–7. 10.1016/S0955-0674(00)00238-6 11454454

[8] Ricci V Galmiche A Doye A Necchi V Solcia E Boquet P. High cell sensitivity to *Helicobacter pylori* VacA toxin depends on a GPI-anchored protein and is not blocked by inhibition of the clathrin-mediated pathway of endocytosis. Mol Biol Cell (2000) 11:3897–909. 10.1091/mbc.11.11.3897 11071915 PMC15045

[9] Lai CH Chang YC Du SY Wang HJ Kuo CH Fang SH Cholesterol depletion reduces *Helicobacter pylori* CagA translocation and CagA-induced responses in AGS cells. Infect Immun (2008) 76:3293–303. 10.1128/IAI.00365-08 18443091 PMC2446742

[10] Wang HJ Cheng WC Cheng HH Lai CH Wang WC. *Helicobacter pylori* cholesteryl glucosides interfere with host membrane phase and affect type IV secretion system function during infection in AGS cells. Mol Microbiol (2012) 83:67–84. 10.1111/j.1365-2958.2011.07910.x 22053852

[11] Lin CJ Liao WC Lin HJ Hsu YM Lin CL Chen YA Statins attenuate *Helicobacter pylori* CagA translocation and reduce incidence of gastric cancer: in vitro and population-based case-control studies. PLoS One (2016) 11:e0146432. 10.1371/journal.pone.0146432 26730715 PMC4701455

[12] Bustin M Lehn DA Landsman D Structural features of the HMG chromosomal proteins and their genes. Biochim Biophys Acta (1990) 1049:231–43. 10.1016/0167-4781(90)90092-G 2200521

[13] Wang H Bloom O Zhang M Vishnubhakat JM Ombrellino M Che J HMG-1 as a late mediator of endotoxin lethality in mice. Science (1999) 285:248–51. 10.1126/science.285.5425.248 10398600

[14] Yang H Wang H Tracey KJ. HMG-1 rediscovered as a cytokine. Shock (2001) 15:247–53. 10.1097/00024382-200115040-00001 11303722

[15] Yang H Wang H Czura CJ Tracey KJ. The cytokine activity of HMGB1. J Leukoc Biol (2005) 78:1–8. 10.1189/jlb.1104648 15734795

[16] Huttunen HJ Kuja-Panula J Rauvala H. Receptor for advanced glycation end products (RAGE) signaling induces CREB-dependent chromogranin expression during neuronal differentiation. J Biol Chem (2002) 277:38635–46. 10.1074/jbc.M202515200 12167613

[17] Sappington PL Yang R Yang H Tracey KJ Delude RL Fink MP. HMGB1 B box increases the permeability of Caco-2 enterocytic monolayers and impairs intestinal barrier function in mice. Gastroenterology (2002) 123:790–802. 10.1053/gast.2002.35391 12198705

[18] Andersson U Wang H Palmblad K Aveberger AC Bloom O Erlandsson-Harris H High mobility group 1 protein (HMG-1) stimulates proinflammatory cytokine synthesis in human monocytes. J Exp Med (2000) 192:565–70. 10.1084/jem.192.4.565 10952726 PMC2193240

[19] Achouiti A van der Meer AJ Florquin S Yang H Tracey KJ van ‘t Veer C High-mobility group box 1 and the receptor for advanced glycation end products contribute to lung injury during *Staphylococcus aureus* pneumonia. Crit Care (2013) 17:R296. 10.1186/cc13162 24342460 PMC4057161

[20] Patel VS Sitapara RA Gore A Phan B Sharma L Sampat V High mobility group box-1 mediates hyperoxia-induced impairment of *Pseudomonas aeruginosa* clearance and inflammatory lung injury in mice. Am J Respir Cell Mol Biol (2013) 48:280–7. 10.1165/rcmb.2012-0279OC 23087050 PMC3604087

[21] Johansson L Snall J Sendi P Linner A Thulin P Linder A HMGB1 in severe soft tissue infections caused by *Streptococcus pyogenes*. Front Cell Infect Microbiol (2014) 4:4. 10.3389/fcimb.2014.00004 24524027 PMC3906589

[22] Radin JN Gonzalez-Rivera C Ivie SE McClain MS Cover TL. *Helicobacter pylori* VacA induces programmed necrosis in gastric epithelial cells. Infect Immun (2011) 79:2535–43. 10.1128/IAI.01370-10 21482684 PMC3191986

[23] Lai CH Kuo CH Chen PY Poon SK Chang CS Wang WC. Association of antibiotic resistance and higher internalization activity in resistant *Helicobacter pylori* isolates. J Antimicrob Chemother (2006) 57:466–71. 10.1093/jac/dki479 16396916

[24] Lu DY Chen HC Yang MS Hsu YM Lin HJ Tang CH Ceramide and toll-like receptor 4 are mobilized into membrane rafts in response to *Helicobacter pylori* infection in gastric epithelial cells. Infect Immun (2012) 80:1823–33. 10.1128/IAI.05856-11 22354030 PMC3347442

[25] Lin CD Kou YY Liao CY Li CH Huang SP Cheng YW Zinc oxide nanoparticles impair bacterial clearance by macrophages. Nanomedicine (Lond) (2014) 9:1327–39. 10.2217/nnm.14.48 24628689

[26] Lin CJ Rao YK Hung CL Feng CL Lane HY Tzeng DT Inhibition of *Helicobacter pylori* CagA-induced pathogenesis by methylantcinate B from *Antrodia camphorata*. Evid Based Complement Alternat Med (2013) 2013:682418. 10.1155/2013/682418 23431343 PMC3562571

[27] Lu DY Tang CH Chang CH Maa MC Fang SH Hsu YM *Helicobacter pylori* attenuates lipopolysaccharide-induced nitric oxide production by murine macrophages. Innate Immun (2012) 18:406–17. 10.1177/1753425911413164 21926162

[28] Lai CH Wang HJ Chang YC Hsieh WC Lin HJ Tang CH *Helicobacter pylori* CagA-mediated IL-8 induction in gastric epithelial cells is cholesterol-dependent and requires the C-terminal tyrosine phosphorylation-containing domain. FEMS Microbiol Lett (2011) 323:155–63. 10.1111/j.1574-6968.2011.02372.x 22092715

[29] Rojas A Gonzalez I Rodriguez B Romero J Figueroa H Llanos J Evidence of involvement of the receptor for advanced glycation end-products (RAGE) in the adhesion of *Helicobacter pylori* to gastric epithelial cells. Microbes Infect (2011) 13:818–23. 10.1016/j.micinf.2011.04.005 21609778

[30] Li G Liang X Lotze MT. HMGB1: the central cytokine for all lymphoid cells. Front Immunol (2013) 4:68. 10.3389/fimmu.2013.00068 23519706 PMC3602962

[31] van Zoelen MA Schmidt AM Florquin S Meijers JC de Beer R de Vos AF Receptor for advanced glycation end products facilitates host defense during *Escherichia coli*-induced abdominal sepsis in mice. J Infect Dis (2009) 200:765–73. 10.1086/604730 19627249

[32] McClellan S Jiang X Barrett R Hazlett LD. High-mobility group box 1: a novel target for treatment of *Pseudomonas aeruginosa* keratitis. J Immunol (2015) 194:1776–87. 10.4049/jimmunol.1401684 25589066 PMC4323849

[33] Harris HE Andersson U Pisetsky DS. HMGB1: a multifunctional alarmin driving autoimmune and inflammatory disease. Nat Rev Rheumatol (2012) 8:195–202. 10.1038/nrrheum.2011.222 22293756

[34] Schiraldi M Raucci A Munoz LM Livoti E Celona B Venereau E HMGB1 promotes recruitment of inflammatory cells to damaged tissues by forming a complex with CXCL12 and signaling via CXCR4. J Exp Med (2012) 209:551–63. 10.1084/jem.20111739 22370717 PMC3302219

[35] Christaki E Lazaridis N Opal SM. Receptor for advanced glycation end products in bacterial infection: is there a role for immune modulation of receptor for advanced glycation end products in the treatment of sepsis? Curr Opin Infect Dis (2012) 25:304–11. 10.1097/QCO.0b013e3283519b82 22327468

[36] Yu M Wang H Ding A Golenbock DT Latz E Czura CJ HMGB1 signals through toll-like receptor (TLR) 4 and TLR2. Shock (2006) 26:174–9. 10.1097/01.shk.0000225404.51320.82 16878026

[37] Schmidt AM Yan SD Yan SF Stern DM The multiligand receptor RAGE as a progression factor amplifying immune and inflammatory responses. J Clin Invest (2001) 108:949–55. 10.1172/JCI200114002 11581294 PMC200958

[38] Sims GP Rowe DC Rietdijk ST Herbst R Coyle AJ. HMGB1 and RAGE in inflammation and cancer. Annu Rev Immunol (2010) 28:367–88. 10.1146/annurev.immunol.021908.132603 20192808

[39] Garlanda C Mantovani A Ligands and receptors of the interleukin-1 family in immunity and disease. Front Immunol (2013) 4:396 10.3389/fimmu.2013.00396 24312101 PMC3834610

[40] Kaczmarek A Vandenabeele P Krysko DV. Necroptosis: the release of damage-associated molecular patterns and its physiological relevance. Immunity (2013) 38:209–23. 10.1016/j.immuni.2013.02.003 23438821

[41] Stephenson HN Herzig A Zychlinsky A. Beyond the grave: when is cell death critical for immunity to infection? Curr Opin Immunol (2016) 38:59–66. 10.1016/j.coi.2015.11.004 26682763

[42] Torok AM Bouton AH Goldberg JB. *Helicobacter pylori* induces interleukin-8 secretion by toll-like receptor 2- and toll-like receptor 5-dependent and -independent pathways. Infect Immun (2005) 73:1523–31. 10.1128/IAI.73.3.1523-1531.2005 15731050 PMC1064968

[43] Chen GY Nunez G. Sterile inflammation: sensing and reacting to damage. Nat Rev Immunol (2010) 10:826–37. 10.1038/nri2873 21088683 PMC3114424

[44] Garlanda C Dinarello CA Mantovani A. The interleukin-1 family: back to the future. Immunity (2013) 39:1003–18. 10.1016/j.immuni.2013.11.010 24332029 PMC3933951

[45] Barry KC Fontana MF Portman JL Dugan AS Vance RE IL-1alpha signaling initiates the inflammatory response to virulent *Legionella pneumophila in vivo*. J Immunol (2013) 190:6329–39. 10.4049/jimmunol.1300100 23686480 PMC3682686

[46] Li C Li H Jiang Z Zhang T Wang Y Li Z Interleukin-33 increases antibacterial defense by activation of inducible nitric oxide synthase in skin. PLoS Pathog (2014) 10:e1003918. 10.1371/journal.ppat.1003918 24586149 PMC3930573

[47] Godlewska R Pawlowski M Dzwonek A Mikula M Ostrowski J Drela N Tip-alpha (hp0596 gene product) is a highly immunogenic *Helicobacter pylori* protein involved in colonization of mouse gastric mucosa. Curr Microbiol (2008) 56:279–86. 10.1007/s00284-007-9083-7 18172719

[48] Shahi H Reiisi S Bahreini R Bagheri N Salimzadeh L Shirzad H. Association between *Helicobacter pylori* cagA, babA2 virulence factors and gastric mucosal interleukin-33 mRNA expression and clinical outcomes in dyspeptic patients. Int J Mol Cell Med (2015) 4:227–34. 27014647 PMC4769600

[49] Entezari M Weiss DJ Sitapara R Whittaker L Wargo MJ Li J Inhibition of high-mobility group box 1 protein (HMGB1) enhances bacterial clearance and protects against *Pseudomonas aeruginosa* pneumonia in cystic fibrosis. Mol Med (2012) 18:477–85. 10.2119/molmed.2012.00024 22314397 PMC3356431

[50] Fox JG Wang TC Inflammation, atrophy, and gastric cancer. J Clin Invest (2007) 117:60–9. 10.1172/JCI30111 17200707 PMC1716216

[51] Ando T Kusugami K Ohsuga M Shinoda M Sakakibara M Saito H Interleukin-8 activity correlates with histological severity in *Helicobacter pylori*-associated antral gastritis. Am J Gastroenterol (1996) 91:1150–6. 8651162

[52] Park JS Arcaroli J Yum HK Yang H Wang H Yang KY Activation of gene expression in human neutrophils by high mobility group box 1 protein. Am J Physiol Cell Physiol (2003) 284:C870–9. 10.1152/ajpcell.00322.2002 12620891

[53] Chung HW Lee SG Kim H Hong DJ Chung JB Stroncek D Serum high mobility group box-1 (HMGB1) is closely associated with the clinical and pathologic features of gastric cancer. J Transl Med (2009) 7:38. 10.1186/1479-5876-7-38 19476625 PMC2694170

[54] Zhang QY Wu LQ Zhang T Han YF Lin X. Autophagy-mediated HMGB1 release promotes gastric cancer cell survival via RAGE activation of extracellular signal-regulated kinases 1/2. Oncol Rep (2015) 33:1630–8. 10.3892/or.2015.3782 25652880 PMC4358082

[55] Morales ME Rojas RA Monasterio AV Gonzalez BI Figueroa CI Manques MB Expression of RAGE in *Helicobacter pylori* infested gastric biopsies. Rev Med Chil (2013) 141:1240–8. 10.4067/S0034-98872013001000002 24522351

[56] Chung HW Jang S Kim H Lim JB. Combined targeting of high-mobility group box-1 and interleukin-8 to control micrometastasis potential in gastric cancer. Int J Cancer (2015) 137:1598–609. 10.1002/ijc.29539 25821182

